# A model and the behavioral implications of the calculus of consent: The dilemma of public choice before public choice

**DOI:** 10.1371/journal.pone.0243728

**Published:** 2020-12-15

**Authors:** Minjung Kim, Do Hyun Park

**Affiliations:** 1 Department of Physics, Sungkyunkwan University, Suwon, Korea; 2 Department of School of Law, Seoul National University, Seoul, Korea; Baylor University, UNITED STATES

## Abstract

The choice of a group decision-making rule is one of the most important political issues. Buchanan and Tullock have provided a framework for analyzing the optimal k-majority rule from the perspective of “methodological individualism.” They proposed the concept of “external costs” and “decision costs” and argued that the optimal k-majority rule takes place where the sum of these two costs–“total costs”–is minimized. Despite the fact that the approach is widely accepted as a tool for dealing with public decision-making rules, the study of formalizing these two costs in a quantitative manner has been relatively rare. We propose a systematic way of modeling these costs considering the assumptions mentioned by Buchanan and Tullock. We find that the resulting shape of the graphs is generally similar to that of the Buchanan-Tullock model, except for some minor details. Then, using this analytical model, we investigate several factors that could affect Buchanan-Tullock’s two costs and the optimal k-majority rule. We show that “clustering of disadvantages” (social factor) and “loss aversion” (personal factor) could increase external costs in Buchanan-Tullock’s model. These factors can result in a separation between the theoretical and actual optimal k-majority rules. Meanwhile, some recent developments in information and communication technologies can not only decrease decision costs, but also increase the same costs simultaneously through amplified “group polarization” (technological factor). If the effect of the former is not the same as that of the latter, this leads to a difference in optimal k-majority rules as well. These discrepancies bring us to the dilemma of “public choice before public choice.”

## Introduction

Since the dawn of history, human beings have formed communities to prevent harm and achieve cooperation. While the decision maker and the affected are the same in individual decision-making, this is not true in group decision-making. Mankind has continued searching for the ideal group decision-making rule. Many communities have chosen “simple majority rule” as the best decision-making method. However, a simple majority rule conflicts with the modern notion of the free and equal human beings, given that all opponents are affected by a bill even when there is just one more proponent [1]. Wicksell pointed out this problem and argued that the desirable group decision-making rule should be “unanimous” [2].

This approach is theoretically desirable, but has two weaknesses in reality. One is its high cost and the other is the room for disagreement by strategic actions. Accordingly, it is necessary to find a different approach. Some thinkers have pursued a way of defining a social welfare function that would be considered ideal from a moral standpoint. Rawls is a prime example. Rawls assumed that when a rational person is in the situation of “veil of ignorance,” he/she must choose “maximin principle” as his/her code of conduct [3]. This principle has a moral character in that it was derived on the assumption that individuals have risk-aversive standard interests. This kind of normalization separates personal preferences in economic areas from those in political areas. Rawls, for example, called the former as “rationality,” and the latter as “reasonableness” [4]. One question that remains here is why the same person has different choice methods for each domain.

Buchanan and Tullock addressed these problems by introducing the concepts of “external costs” and “decision costs” in *The Calculus of Consent* [5]. They defined external costs as those incurred by group decision-making when some people did not agree on it. Meanwhile, decision costs are associated with the time and effort required to reach a consensus. A k-majority rule decides the minimum number of people who need to vote “yea” to pass a proposal. The aforementioned costs are the functions of the *k* value. Like any other cost-based approach in economics, Buchanan and Tullock insisted that the optimal k-majority rule should be at the point where the value of “(social) interdependent costs” or “total costs” for short, which is the sum of external and decision costs, is minimized. In other words, this optimal point is where the absolute value of the marginal external costs (*MEC*) equals that of the marginal decision costs (*MDC*). Otherwise, when we change the k-value, a decrease in one cost is different from the increase in the other, which means it is not at the optimal point.

Buchanan and Tullock thought that if all community members agree with the best k-majority rule *ex ante*, the contradiction between simple majority and unanimity rules can be resolved. This is because enacting legislation does not necessarily have to follow a unanimous rule while achieving a unanimous agreement under a constitution. In addition, unlike Rawls, they thought that the members of a community do not necessarily adopt a single social welfare function. Departing from the moral and metaphysical assumptions, Buchanan and Tullock respected the diverse preferences of each individual. Their idea that we should decide public choices not through a holistic view but by an aggregation of individual preferences is called “methodological individualism.” They stressed that if liberal and rational individuals exchange their preferences in each legislation by “log-rolling,” which means the trading of votes, the final state may be Pareto optimal and there is no discrepancy between economic and political rationalities. The study of non-market decision-making, including political one, using economic methodologies is called “public choice theory” [6]. The two authors have conducted a number of studies in this field, reflecting the implications of *The Calculus of Consent* since they wrote the book. In particular, James M. Buchanan won the Nobel Prize in economics in 1986 for his contribution to public choice theory [7–10].

Since the pioneering work of Buchanan and Tullock, researchers have conducted a number of studies on the theory of public decision-making rules. Kafoglis and Cebula extended the Buchanan-Tullock model to consider group size and preference heterogeneity [11]. Boudreaux and Lipford applied Buchanan-Tullock’s model and its extensions to the analysis of the voting rules of the European Union [12]. Guttman pointed out human fallibility, and showed that the function of Type I and Type II errors can make the shape of external costs and the optimal k-value different from those of Buchanan-Tullock’s model [13]. However, none of the previous works yielded analytical results, focusing instead mainly on graphical descriptions. Recently, Dougherty and Edward obtained external and decision cost functions shaped as a logit function, which is also the shape of the probability of passing proposals [14, 15]. In addition, more realistic situations in which every individual trying to enact a bill belongs to one of multiple groups with different utility functions have been examined [16]. The authors obtained results that were mostly consistent with the previous ones by Dougherty and Edward [14, 15]. These results depend on a parameter, “per round decision cost,” which is the unit of time and effort measured in decision costs. Thus, this parameter determines the relative weight of external and decision costs, which has a tremendous effect on the final optimal k-majority rule. A more systematic approach, whose results do not largely depend on such a specific parameter, seems to be needed.

In this paper, we propose a systematic formulation of external and decision costs in a more unified manner. To this end, we note that decision costs are proportional to the probability of not passing a proposal. Decision and external costs are symmetric because the latter are proportional to the probability of passing a proposal. We add one more component to this here. Some factors affecting these costs that Buchanan and Tullock did not reflect in their model will be considered in our model in the following discussion.

Buchanan and Tullock also recognized the existence of these factors, but probably thought that their effects would cancel each other out. This idea is also a general concept of neoclassical economics. However, if each of them is not a simple error (noise) but a kind of systemic human behavior (bias), we should consider it in modeling public decision-making rules. Adding these factors to Buchanan-Tullock’s model provides a more realistic optimal k-majority rule, which is different from the one in their original model. In this regard, we discuss some examples in terms of the two costs. First, we will examine the “clustering of disadvantages” (social factor) and “loss aversion” (personal factor), regarding external costs. Second, we will examine some recent rapid developments in information and communication technologies (ICTs) and following amplified “group polarization,” in terms of decision costs (technological factor). We point out that the separation of the optimal points brings us to the dilemma of “public choice before public choice.”

## Modeling framework

Let us consider a group of *N* individuals trying to design a public decision-making rule. The rule determined at the constitutional stage affects all of the votes during the parliamentary stage. It is assumed that all individuals are so rational that they agree on the optimal k-majority rule, where total costs are minimized. This presupposition is one of the core assumptions of Buchanan and Tullock’s theory. For convenience of discussion, the optimal point is assumed to be single.

In our model, all people offered a specific bill have two alternatives. It is assumed that they favor the proposal with the probability of *p* or refuse to accept it in favor of the status quo with the probability of *1*–*p*. We suppose a situation in which voters do not know specifically whether they would approve or reject each bill at the constitutional stage, and the only thing they know is its probability distribution. This is named as “veil of uncertainty” by Buchanan and Tullock. Strictly speaking, they did not mention exactly what the meaning of “uncertainty” was in *The Calculus of Consent*. In economics, uncertainty refers to the state in which an actor is fully ignorant or roughly knows the probability distributions [17]. We choose the latter here because if not, we must interpret the well-known concept of “rational ignorance” only as the situation in which all actors have no information at all. It seems that “ignorance” is not regarded as the situation in which every actor has the same level of information as there being no information at all [18]. Applying this interpretation, all voters may take on a single expected cost function at the constitutional stage, while having different preferences at the parliamentary stage. This is mathematically the same as the situation in which all individuals vote stochastically on decision-making rules at the constitutional stage. For now, we assume that *p* is a constant at 1/2 in our current model. This means no *ex ante* information, which is “the principle of equal a priori probability.” This supposition will be further refined and generalized below.

For this social system, the number of “yea” votes follows the binomial distribution given by

Pr(s)=CNsps(1−p)N−s. (1)

where *s* is a random variable representing the number of “yea” votes in *N* Bernoulli trials. Then, the probability that the proposal will pass under a given k-majority rule can be written as

Prp(k)=∑s=kNCNsps(1−p)N−s. (2)

The probability of not passing the proposal can be expressed in the same way as in Eq (2) by

Prr(k)=∑s=0k−1CNsps(1−p)N−s. (3)

**Fig 1:**
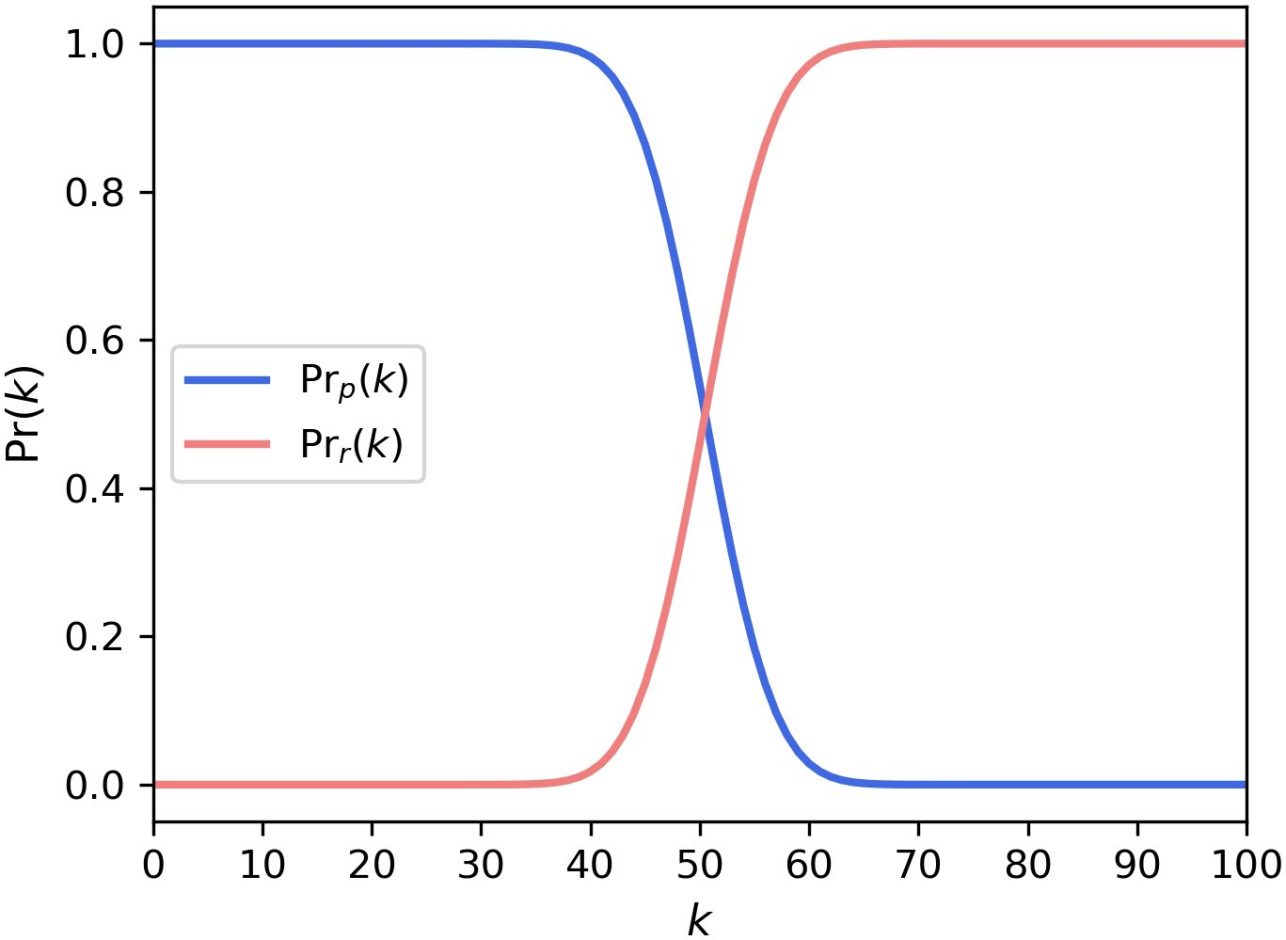
The probability of passing the proposal and not passing the proposal. Probabilities were calculated with *p* = 0.5, *N* = 100.

These two probabilities are shown in Fig 1, in which the variations of the probabilities with k-majority rules are plotted. Unless otherwise noted, the number of individuals is set to 100. We find that both curves are shaped like a logit function.

To be more exact, “external costs” and “decision costs” are expected values because they occur after the votes, not at the rule-making stage. In this regard, Dougherty and Edward [14] defined “expected external costs” as the expected number of opponents per voter when our society decides to enforce a bill. Expected external costs occur only when there is more than one opponent against the proposal passed. Therefore, if every voter approves, there is no expected external cost. By extending this concept, we define “expected decision costs” as the expected number of opponents per voter who must be further persuaded to allow a bill to pass under the given k-majority rule. Expected decision costs occur only when there is more than one proponent for the proposal to be passed. Thus, if all voters disapprove, there is no expected decision cost. These costs are then given, respectively, by

ECe(k)=1N∑s=kN−1CNsps(1−p)N−s(N−s), (4)

ECd(k)=1N∑s=1k−1CNsps(1−p)N−s(k−s). (5)

Here, we assume that the relative weight of the expected external and decision costs is the same. Under some circumstances, an individual might place more weight on one cost than on the other. However, such a change does not affect the overall logic structures, except for a few details.

**Fig 2:**
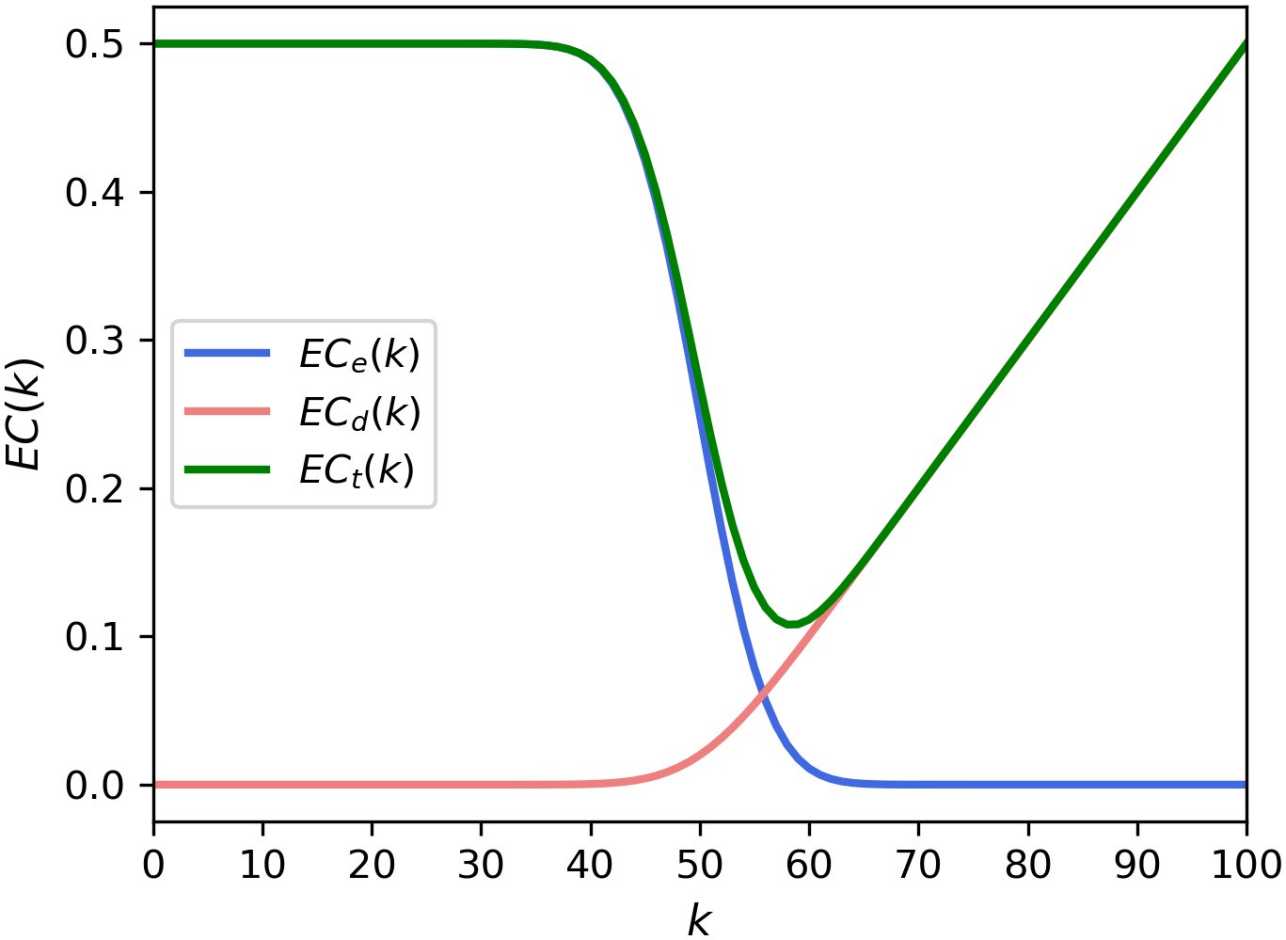
Expected costs from theoretical expressions in Eqs (4) and (5) of the present model. The expected costs were calculated with *p* = 0.5, *N* = 100.

The two costs are shown in Fig 2. We can see that the shape of the expected external costs and probability of passing the proposal is like a logit function. On the other hand, expected decision costs almost become linear for *k* > 50, so that it is no longer a logit function. As a result, the value of the expected total costs, the sum of expected external and decision costs, is minimized at *k* = 58.

These formulas are not perfect because everyone’s costs are considered to have an equal unit value. Under this condition, the assumption of diverse preferences introduced by Buchanan and Tullock is nullified. In the real vote, there may be a difference in the intensity of preferences for each proposal. Therefore, we now examine several subtleties mentioned by Buchanan and Tullock that the model from the binomial distribution did not reflect. Suppose a specific proposal with the k-majority rule has been passed. In Eq (4), we assumed that only the expected number of opponents per voter determined the expected external costs. This means that under the more inclusive k-majority rule, the lower external costs are incurred. In addition, the more people agree on an alternative, the less likely the choice is irrational because of so-called “collective intelligence” [19]. Thus, we introduce a new parameter, *e*_*1*_, to show this effect. The expected external costs with the additional parameter *e*_*1*_ can be expressed as

ECe(k)=1N∑s=kN−1CNsps(1−p)N−s(N−s)e1N−kN. (6)

The parameter *e*_*1*_ has a constant value greater than or equal to 1, and if *k* = *N*, the entire term containing *e*_*1*_ equals 1 (perfect collective intelligence). As we saw earlier, there is no expected external cost in this case.

Now, consider the opposite situation of persuading those who disagree to pass the same bill under the given k-majority rule. The smaller the number of remaining opponents, the more difficult it will be to persuade each of them. This is because, like game theory, we think and act strategically. However, if the k-value is low, there are many candidates to persuade, so their power or the possibility of strategic behavior is not that high. Therefore, we introduce an additional parameter *d*_*1*_ to reflect it. The expected decision costs with parameter *d*_*1*_ can be represented as

ECd(k)=1N∑s=1k−1CNsps(1−p)N−s(k−s)d1kN. (7)

The parameter *d*_*1*_ also has a constant value greater than or equal to 1, and if *k* = 0, the entire term containing *d*_*1*_ equals 1 (non-strategic situation). Similar to the previous analysis, there is no expected decision cost in this case.

**Fig 3:**
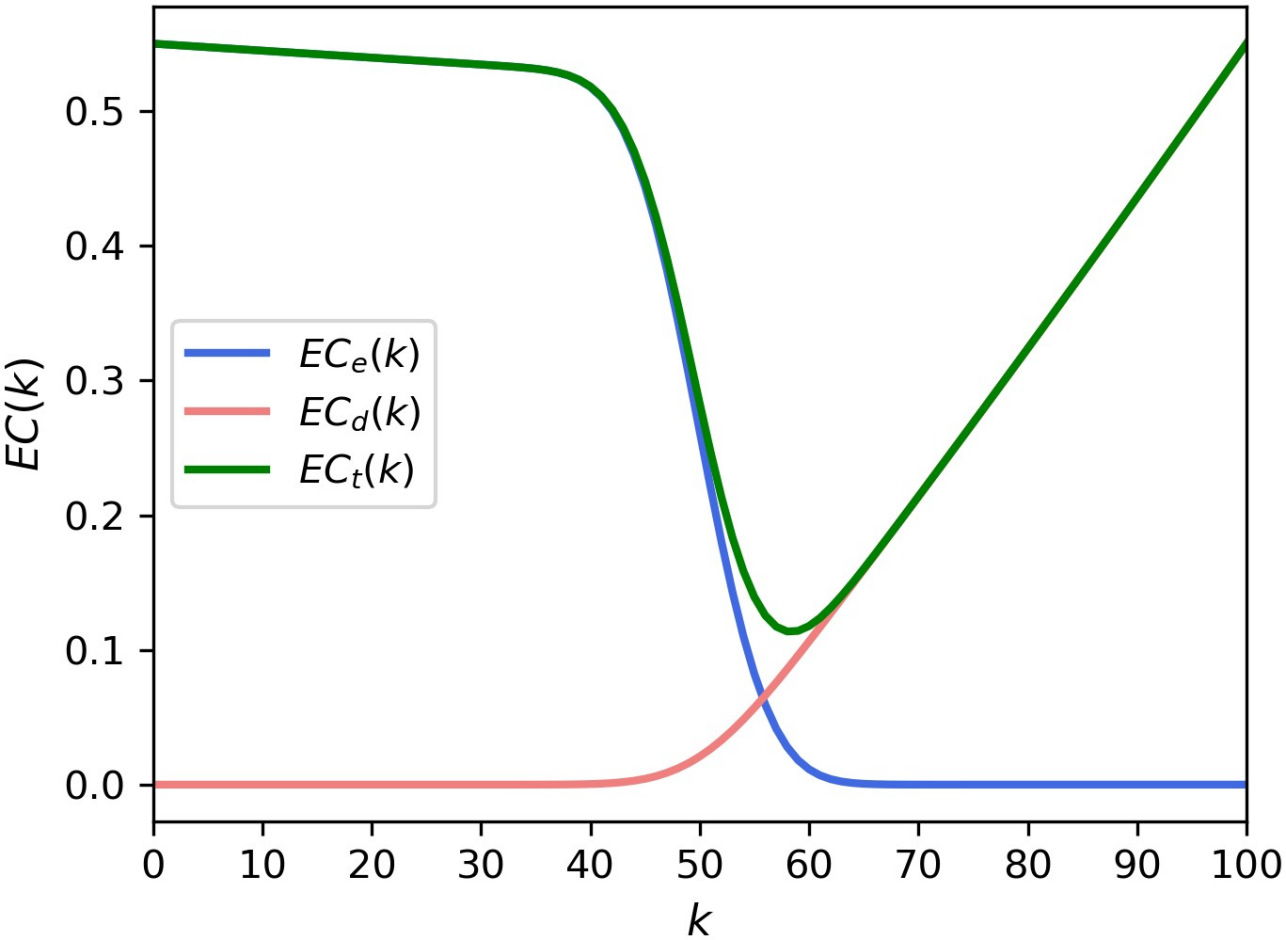
Expected costs from theoretical expressions in Eqs (6) and (7) of the present model. The expected costs were calculated with *p* = 0.5, *N* = 100, and *e*_*1*_ = *d*_*1*_ = 1.1.

With the refining parameters included, the two costs from Eqs (6) and (7) are shown in Fig 3. It can be seen that the value of expected external costs, which is a constant when approximately *k* < 40 in Fig 2, decreases as *k* increases, as shown in Fig 3. The optimal point is *k* = 58, which is the same as the one in Fig 2. *e*_*1*_ and *d*_*1*_ were set to 1.1.

A similar but separate logic, as we introduced in Eqs (6) and (7), can be applied to cases even under the same k-majority rule. Assuming that the k-value is a constant such as 90 and the ratio of proponents is 91% or 92%, the effects of collective intelligence are not the same. This result is different from the effect of the k-change itself. This is similar to the Condorcet Jury theorem, which states that if all jurors are making the right choice just more than 50 percent, the more jurors vote, the lower the error of the group decision-making is [20]. Likewise, the probability of strategic behaviors (e.g., holding-out) is not the same; for example, when the k-value is 90 and the ratio of opponents is 11% or 12%. This is also not the same as the effect of the k-change itself. The two parameters *e*_*2*_ and *d*_*2*_ are used to represent these effects on the external and decision costs, respectively, as

ECe(k)=1N∑s=kN−1CNsps(1−p)N−s(N−s)e2N−sNe1N−kN, (8)

ECd(k)=1N∑s=1k−1CNsps(1−p)N−s(k−s)d2sNd1kN. (9)

**Fig 4:**
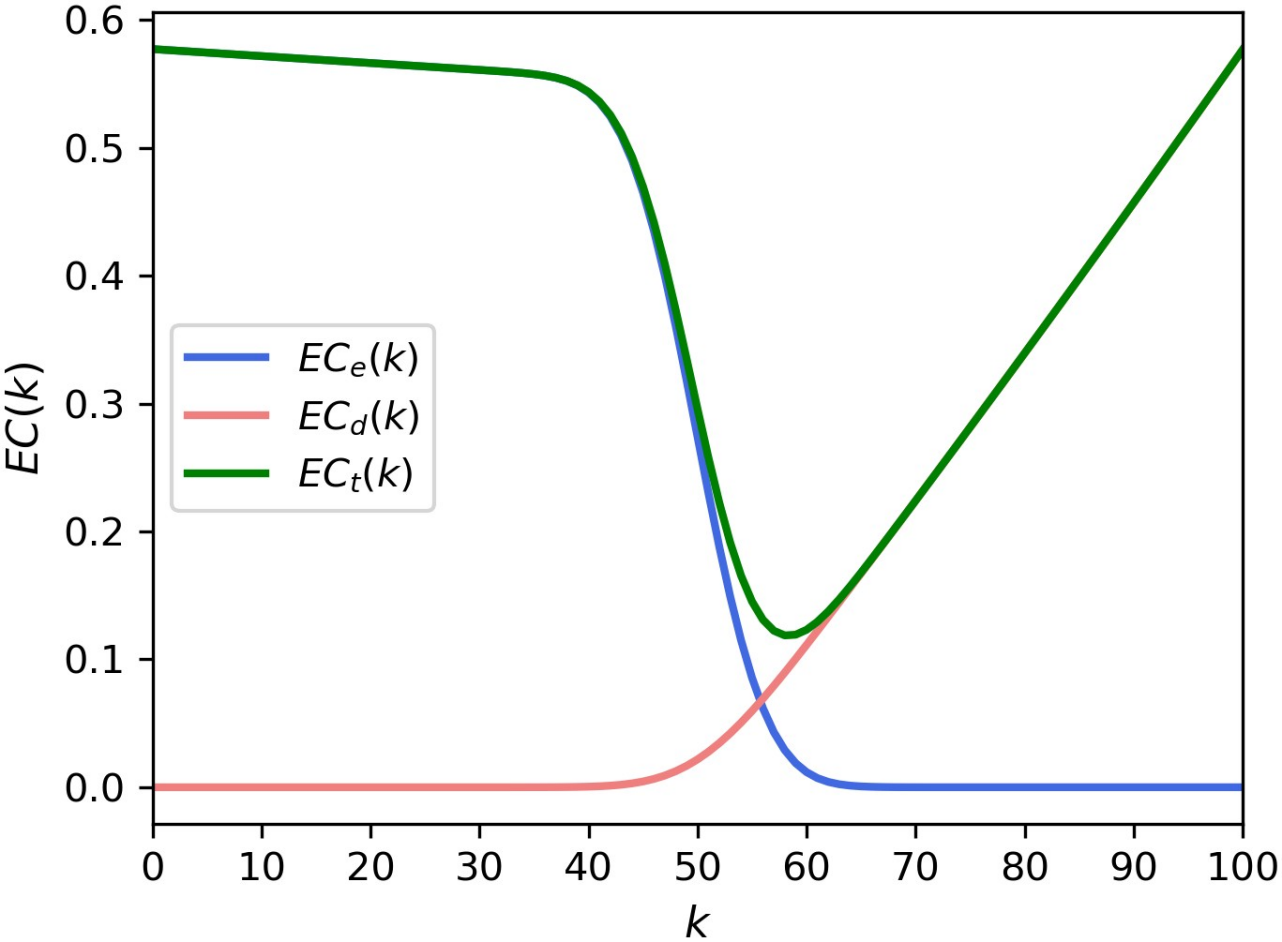
Expected costs from theoretical expressions in Eqs (8) and (9) of the present model. The expected costs were calculated with *p* = 0.5, *N* = 100, and *e*_*1*_ = *d*_*1*_ = *e*_*2*_ = *d*_*2*_ = 1.1.

Fig 4 presents the theoretical cost curves from Eqs (8) and (9). We set the values of the parameters as *e*_*2*_ = 1.1, and *d*_*2*_ = 1.1. The resultant curves in Fig 4 are generally similar to the shapes of the graphs in Fig 3. The optimal k-value is 58, which is the same as that in Figs 2 and 3.

Now, we will generalize our model assuming that voters can have different probability distributions depending on the quantity and quality of information that they have at the constitutional stage. Each community may have different levels of information, but all members in the same community share similar contents. We also assume that the utility is a random variable and has a different value for each extraction. In this way, individual expected utility can be modeled with a certain distribution, and each utility is extracted from the given distribution function. No matter what type of probability distribution you choose, it is in line with our discussion. If each extraction is independent, individual preferences at the parliamentary stage are not always the same. This assumption of “independent and identically distributed” virtually describes the internal and external independence of preferences in neoclassical economics. In addition, this attempt does not fundamentally violate the assumption of “veil of uncertainty.” In principle, simulation methods should be used for this approach. However, to keep it simple, we use mean values from distributions to reflect these effects in analytical expressions.

Let us consider a case in which each member *i* of society has his/her utility *μ*_*i*_ ranging from 0 to 1. The utility is randomly drawn from a predetermined distribution. The larger the utility is, the greater the tendency to agree with the proposal is. If an ith individual’s utility is greater than 0.5, he/she will be in favor of the proposal. Otherwise, he/she will choose to maintain the status quo. We thus obtain the probability of the ith individual’s vote for yea as

$$p_{i}=\{\begin{array}{cc} 1 & \mu_{i}\geq 0.5\\ 0 & \mu_{i}<0.5 \end{array}.$$ (10)

The number of individuals voting for yea is then given by

$$R=\sum_{i=1}^{N}p_{i}.$$ (11)

We now redefine the expected external costs using an individual’s utility. Let us consider individual *i*, whose utility is *μ*_*i*_. He/she would perceive his/her losses caused by passing a proposal as 1 − *μ*_*i*_. Even those who approve the bill could expect this type of loss according to their own utilities. From this point of view, we propose redefining expected external costs as expected losses per voter. Instead of using each realized utility from repetitive simulations, we approximate utilities with the mean value of some types of distribution functions. To distinguish the utilities of proponents from those of opponents about an issue, we calculate the mean values of their utilities separately. Although this approximation seems to be crude, we note that the errors would be negligible as the number of individuals increases.

Likewise, we can redefine the expected decision costs. The ideal state of proponents is achieved when *μ*_*i*_ equals 1. Except for it, even those who approve a bill need to be more convinced to be perfect proponents. Thus, we can redefine expected decision costs as the average losses (losses in comparison with the ideal condition) per voter. It is 1 − *μ*_*i*_ once again. The expected external and decision costs can be expressed as

ECe(k)=1N∑s=kN−1CNsps(1−p)N−s{l¯o(N−s)+l¯ps}e2N−sNe1N−kN, (12)

ECd(k)=1N∑s=1k−1CNsps(1−p)N−s{l¯o(k−s)+l¯ps}d2sNd1kN, (13)

where ${\bar{l}}_{o}$ and ${\bar{l}}_{p}$ are the mean losses for opponents and proponents, respectively, which are calculated as

$${\bar{l}}_{o}=\frac{1}{\left(0.5-0\right)}\int_{0}^{0.5}\left(1-\mu \right)f(\mu )d\mu ,$$ (14)

$${\bar{l}}_{p}=\frac{1}{\left(1-0.5\right)}\int_{0.5}^{1}\left(1-\mu \right)f(\mu )d\mu .$$ (15)

Here, *f*(*μ*) is an arbitrary distribution function for utility.

**Fig 5:**
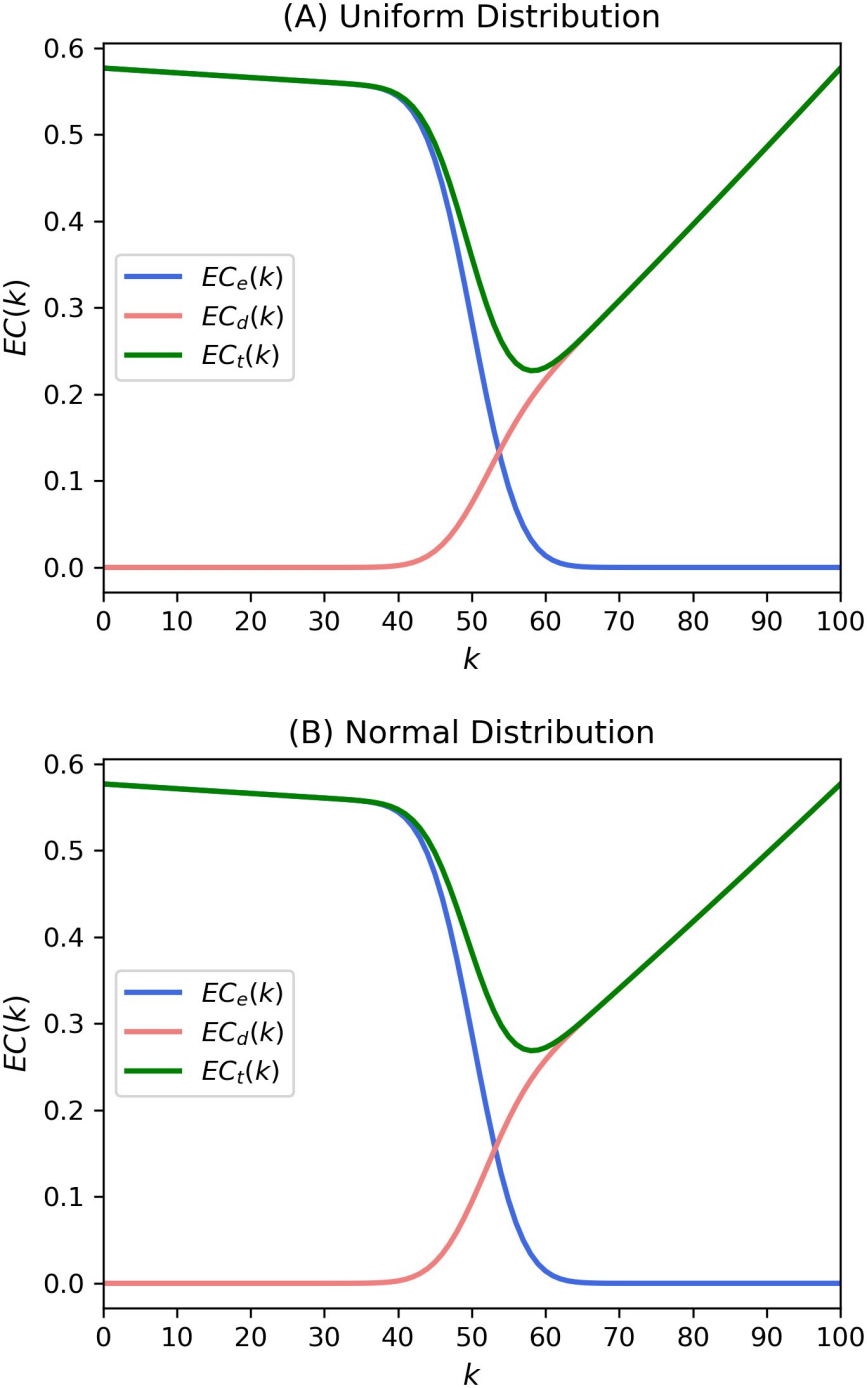
Expected costs from theoretical expressions in Eqs (12) and (13) of the present model with utility distribution from (A) uniform distribution and (B) normal distribution. The expected costs were calculated with *p* = 0.5, *N* = 100, and *e*_*1*_ = *d*_*1*_ = *e*_*2*_ = *d*_*2*_ = 1.1.

We perform some calculations on two types of utility distributions: uniform and normal distributions. First, consider a uniform distribution that is defined for 0 ≤ *μ* ≤ 1. The mean losses ${\bar{l}}_{o}$ and ${\bar{l}}_{p}$ are calculated as 0.75 and 0.25, respectively. The resultant cost curves from Eqs (12) and (13) are shown in Fig 5A. The other case in which the utility is drawn from a normal distribution with mean 0.5 and standard deviation 0.2 is illustrated in Fig 5B. The normalization constant for the normal distribution was adjusted to confine the range of *μ* to 0 ≤ *μ* ≤ 1. Mean losses for opponents ${\bar{l}}_{o}=0.6545$ and mean losses for proponents ${\bar{l}}_{p}=0.3455$. The graphs are similar for both uniform and Gaussian utility distributions. In both cases, the optimal *k* value is 58, which is the same as that in Figs 2–4.

We can see that the results of the present model are generally in good agreement with those of Buchanan and Tullock’s theory. Specifically, in our model, as the k-value increases, the external cost function decreases, the decision cost function increases, and the shape of the total cost function is convex. These are the essential characteristics of Buchanan and Tullock’s model. In addition, as we mentioned, our model is derived not from simulations but from analytic results. Although our model is approximated in some cases and slightly complicated by introducing some parameters, the core of Buchanan and Tullock’s model remains and it does not depend on a specific single parameter.

## Implications

Several factors affecting the two costs of group decision-making can be examined using the new analytical model introduced in the previous discussion. In the following section, we examine two factors regarding external costs and one factor regarding decision costs. The former concerns the clustering of disadvantages (social factor) and loss aversion (personal factor) that can affect external costs, which can lead to the difference between theoretical and actual optimal k-majority rules. The latter concerns the conflicting impact of ICT (technological factor) that can affect decision costs, which can result in the same kind of difference between theory and reality.

### Clustering of disadvantages on costs

All individuals belong to groups with different influences on a variety of social problems. Buchanan and Tullock supposed that each voter is frequently involved in distinct interest groups for each issue, so that one voter may have little power in one proposal, but he/she can have greater influence in another. They thought these effects would cancel out each other by mutual political agreements known as “log-rolling.” Is that true in reality? Rather, once the “veil” is removed, groups that exert a strong influence on one agenda are more likely to exert significant influence on other issues. Therefore, an individual who once belonged to a low-power group continues to predict that his/her opinion will be opposed to that of society. Wolff and De-Shalit expressed this as the concept of “clustering of disadvantages” [21]. If this effect exists in reality, the external costs in these groups would systemically rise. This is because unexpected additional costs by inequality are created. We express this consideration mathematically in the following way:

ECe(k)=1N∑s=kN−1CNsps(1−p)N−s{l¯o(N−s)+l¯ps}e2N−sNe1N−kN×ec. (16)

**Fig 6:**
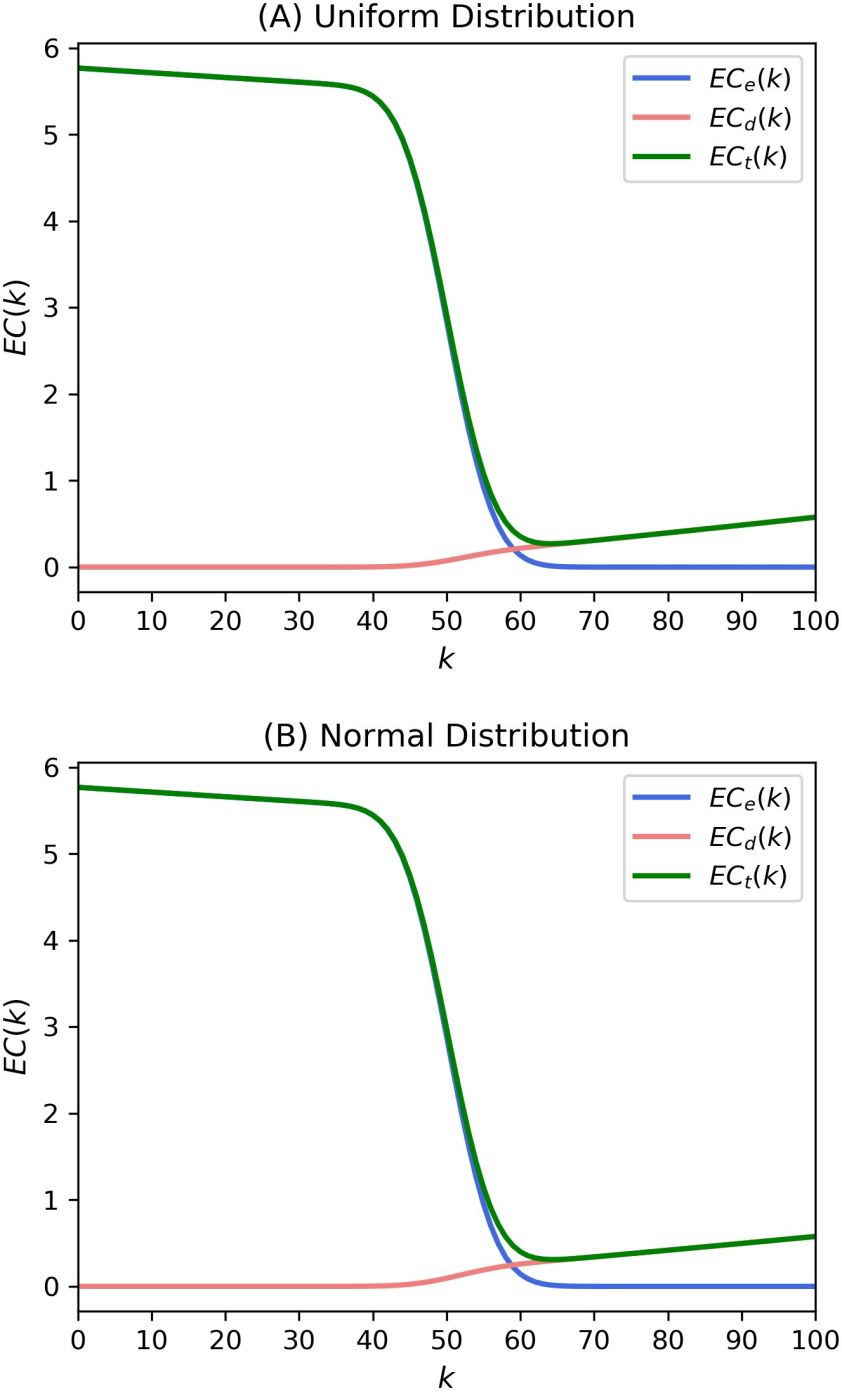
Expected costs from theoretical expressions in Eqs (13) and (16) of the present model with utilities from (A) uniform distribution and (B) normal distribution. The expected costs were calculated with *p* = 0.5, *N* = 100, *e*_*1*_ = *d*_*1*_ = *e*_*2*_ = *d*_*2*_ = 1.1, and *e*_*c*_ = 10.

Eq (16) is based on Eq (12). We introduce a new parameter *e*_*c*_ to represent the extent of the clustering of disadvantages effect. The parameter greater than 1 gives an additional weight to external costs. In Fig 6A and 6B, the results of Eqs (13) and (16) for the cases of utilities following uniform and Gaussian distributions are shown, respectively. We can see that the optimal k-value and the minimum value of total costs increase compared to the results of Eqs (12) and (13) in both cases. The optimal k increased from 58 to 64 in both Fig 6A and 6B. This means that the “theoretical” expected external costs from Eq (12) and “actual” ones from Eq (16) are different from each other in most situations. This leads us to the dilemma that we should make a “public choice” between theory and reality before making the rule of public choice.

### Loss aversion when society makes public decisions

We have treated optimal public decision-making rules under the assumption that all voters are “rational.” Buchanan and Tullock did not specify exactly what rational decision-making meant, and the conception of “rationality” can be considered in several ways. First, we may take the view of “homo economicus” which is commonly used in the public choice theory. It is also a typical decision-maker in neoclassical economics. Homo economicus has “perfect” rationality, willpower, and self-interest so that he/she does not regret his/her own decision [22]. This means that his/her willingness to pay (*WTP*) in decision-making is always the same as willingness to accept (*WTA*) of the choice. There is one major problem with this: Homo economicus always has all the information regarding his/her choices. However, this contradicts Buchanan and Tullock’s aforementioned assumption of “veil of uncertainty.” In addition, if we assume that he/she has perfect information, the concept of “rational ignorance,” which is widely used in the field of public choice, becomes useless. To avoid this problem, we use the concept of rationality as “bounded rationality” in the field of behavioral economics. Behavioral economists have found that humans do not usually have perfect information, and often depend on heuristics (system 1), which could lead us to cognitive bias [23]. Bias is a distinct concept from simple errors (noise), because bias appears systematically in mankind and cannot simply be canceled out. In this regard, Kahneman and Tversky presented a pioneering model of “prospect theory” [24]. In their model, people have a tendency to put more weight on losses than gains. It is different from the theory that neoclassical economics supports, “expected utility theory,” and it is known as “loss aversion.” If we, real humans, cannot escape from loss aversion, the value of WTA will be systemically more than that of WTP. A subsequent study proved a separation between WTP and WTA in this way [25]. Note that bounded rationality is not related to the imperfectness of information acquisition, but to the imperfectness of information processing. The former also applies to homo economicus when there is asymmetric information, which is one factor of market failure.

Therefore, the irrational aspect of human behaviors that we have not considered in our model could make a difference. Let us consider an example of land expropriation. When a government decides to expropriate private land, the owner of the land can demand just compensation which is stipulated in related laws. However, in this situation, what is “just compensation”? If we accept the existence of loss aversion, the land owner may think that just compensation means WTA. However, the others who vote for that the land should be expropriated maybe regard just compensation as WTP. The optimal k-majority rule Buchanan and Tullock derived is divided into the “subjective” optimal rule that the owner of land supports and the “objective” one that the others support. In our model, the first idea leads to the effect of raising external costs overall when the government passes a specific bill. It can be expressed as

ECe(k)=λ[1N∑s=kN−1CNsps(1−p)N−s{l¯o(N−s)+l¯ps}e2N−sNe1N−kN]α. (17)

where *λ* and *α* are parameters controlling the loss aversion effect. The values of the parameters used are *α* = 0.88 and *λ* = 2.55, estimated by Tversky and Kahneman [26].

**Fig 7:**
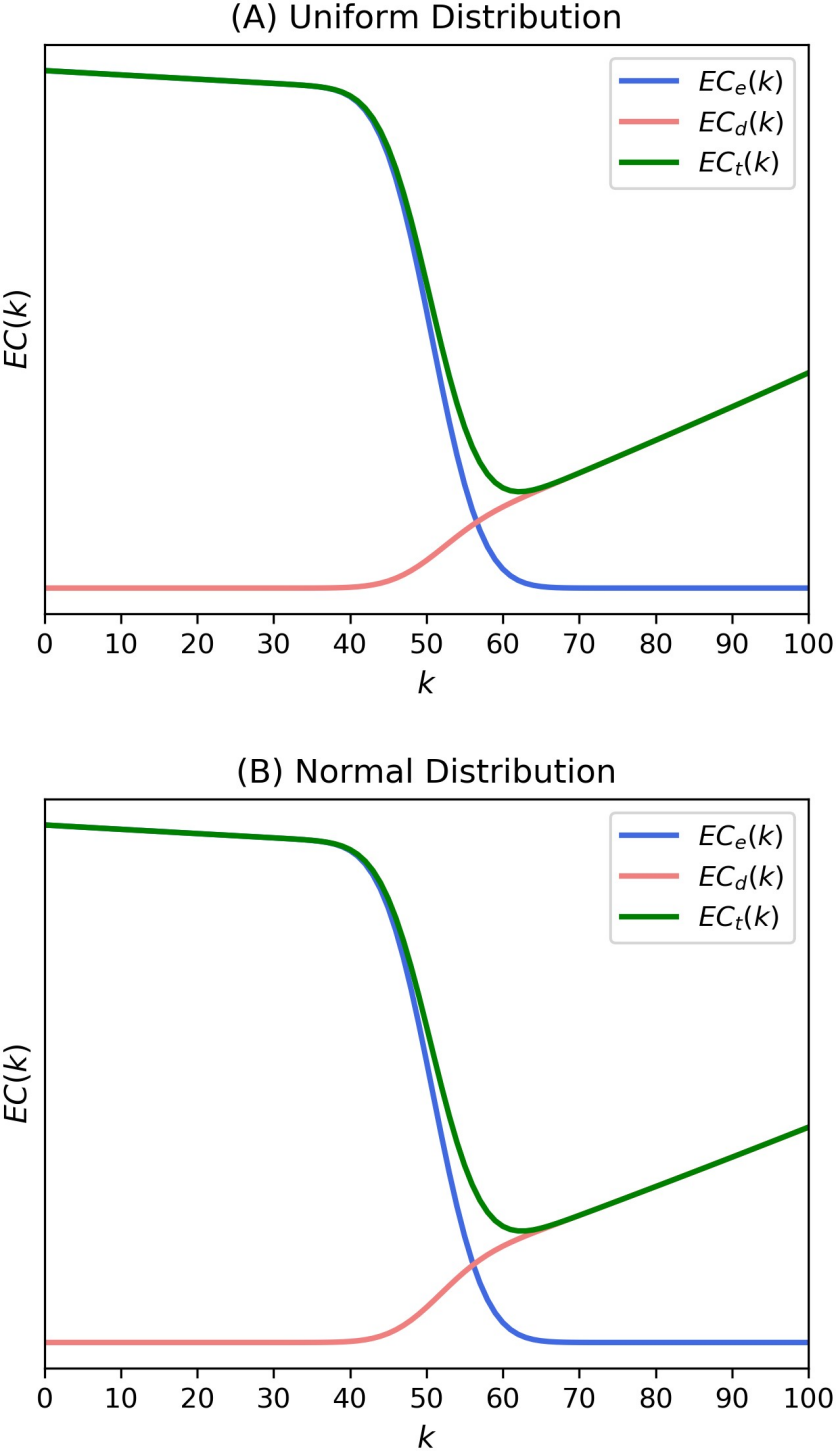
Expected costs with loss aversion from theoretical expressions in Eqs (13) and (17) of the present model with utilities from (A) uniform distribution and (B) normal distribution. These expected costs were calculated with *p* = 0.5, *N* = 100, and *e*_*1*_ = *d*_*1*_ = *e*_*2*_ = *d*_*2*_ = 1.1. The plot uses *α* = 0 and *λ* = 2.25.

A graphical representation of this effect is illustrated in Fig 7. Fig 7A and 7B depict the expected costs for the utilities following uniform and Gaussian distributions, respectively. We can see higher external and total costs, and a subsequent more inclusive k-majority rule compared to the results of Eqs (12) and (13) in Fig 5. The k-value increased from 58 to 62. This systemic increase of expected external costs by loss aversion also leads us to the same discrepancy that we showed in the previous discussion.

### Conflicting impacts of ICTs and group polarization

Vragov and Kumar investigated the effect of information and communication technologies (ICTs) on the costs of group decision-making [27]. These authors employed Buchanan and Tullock’s model, including a technology variable, in which they derived some policies to reduce decision and total costs. They found that increased use of ICT tools can improve multilateral communication and negotiation, which reduces decision costs and changes the optimal k-majority rule in some situations. In conclusion, they proposed two policies: more investment in technologies and choosing an optimal k-majority rule.

However, much uncertainty still exists about the relationship between technologies and the effects of communication among citizens. Recent advances in ICTs have not only facilitated better negotiations but also brought about more polarization of group opinions than ever. This effect is well-known as the “echo chamber” or “filter bubble” [28–30]. The amplified “group polarization” from state-of-the-art ICTs could increase the time and effort required to reach a consensus, which leads to an increase in expected decision and total costs. Therefore, improvements in ICTs have two contradictory consequences. They can decrease decision costs by making multilateral communication and negotiation easier. Meanwhile, they also cause several negative phenomena such as fake news as well as echo chamber and filter bubble, making it difficult to reach a consensus. As a result, decision and total costs depend on the magnitude of these two conflicting effects. We propose the expression considering these effects as follows:

ECd(k)=1N∑s=1N−1CNsps(1−p)N−s{l¯o(k−s)+l¯ps}d2sNd1kN×dp−di. (18)

**Fig 8:**
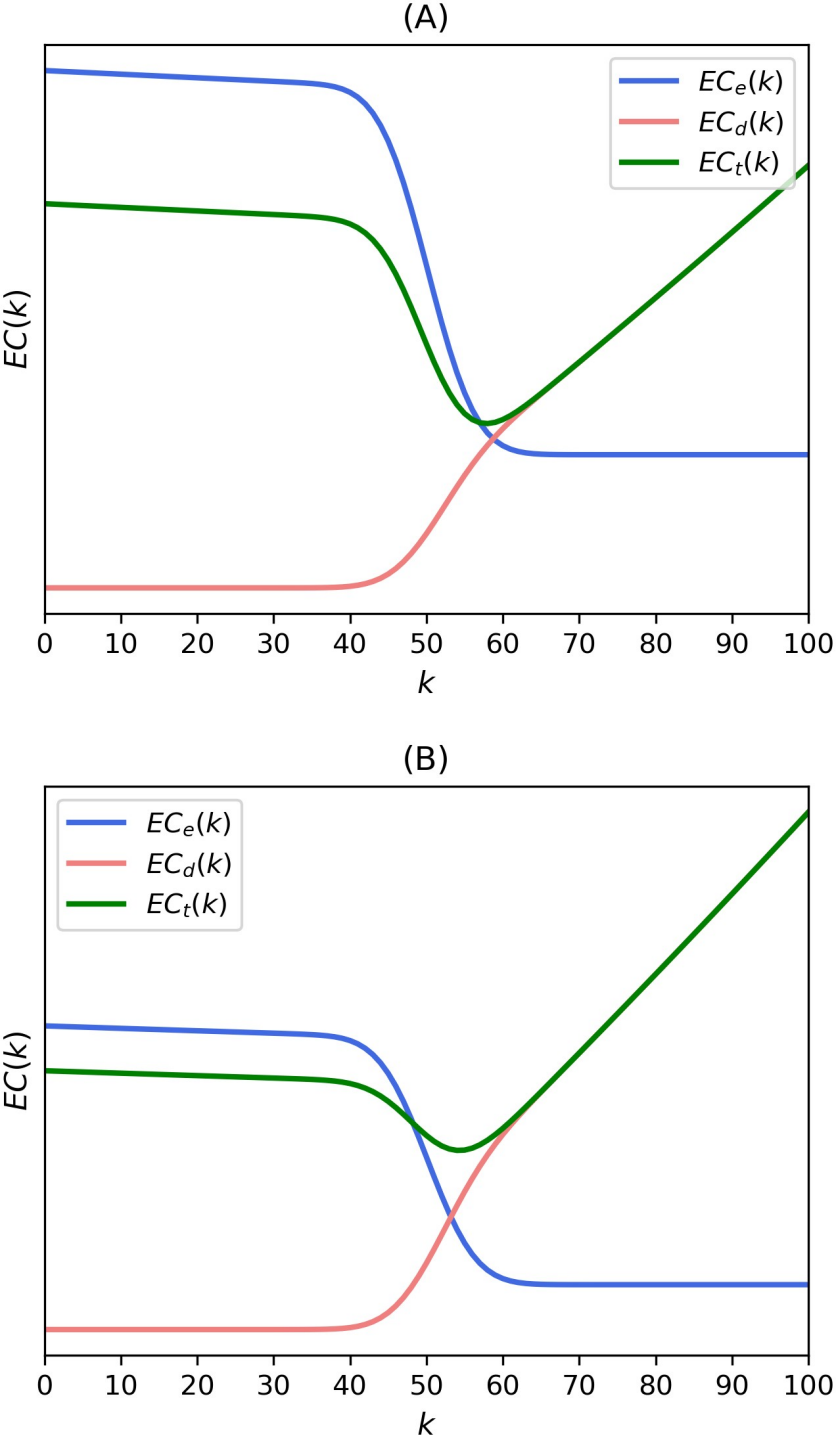
The Effects of technology advancements on expected decision and total costs with uniform distribution when (A) dialogue effect is greater than polarization effect and (B) polarization effect is greater than dialogue effect. The expected costs were calculated with *p* = 0.5, *N* = 100, *e*_*1*_ = *d*_*1*_ = *e*_*2*_ = *d*_*2*_ = 1.1, and (A) *d*_*p*_ = 1.1, *d*_*i*_ = 0.2 (B) *d*_*p*_ = 2, *d*_*i*_ = 0.1.

We define *d*_*i*_ as the “dialogue effect,” which symbolizes the effect that much communication makes us more cooperative, and *d*_*p*_ as “polarization effect,” which symbolizes the effect that excessive discussion confined to only the same-minded groups makes us more prejudiced. If the former effect is greater than the latter, the decision cost function shifts to the right, and the optimal k-value increases as depicted in Fig 8A. In this case, the value of k is 58. On the contrary, if ICT makes reaching an agreement more difficult, the decision cost function shifts to the left, which leads to the lower k-majority rule. This process is illustrated in Fig 8B. The optimal k-value decreases from 58 to 54. Both Fig 8A and 8B represent the cases of uniform utility distributions for the convenience of discussions. We face the same separation of the optimal k-majority rules in between theory and reality once again.

## Conclusion

We have introduced a new theoretical framework for calculating the expected external and decision costs associated with public decision-making. Our analytical model is based on a binomial formula and supposes that the two costs are proportional to the number of members associated with a specific proposal. We have confirmed that our theoretical model yields results that are similar to those of the Buchanan-Tullock model in a number of situations. The main advantage of our new method is that it does not depend on a specific parameter like previous studies did. Our model is also advantageous for introducing multiple variables that represent assumptions not covered in previous works. At the same time, our method is analytic, so it does not require any simulation methods. Although we approximated by using mean values of continuous utility distributions to incorporate continuous distribution into analytical models, the benefits of sophisticated quantitative group decision-making models can outweigh such a small shortcoming. Further research could be conducted to test and improve our findings in the real world.

In addition, we applied our framework to a couple of examples of reality. Buchanan and Tullock’s theory did not clearly mention the meaning of “rationality,” so we can consider various conceptions of it. Then, there can be separations between theoretical and actual public choices. In terms of external costs, we found that the existence of clustering of disadvantages (social factor) and loss aversion (personal factor) makes external and total costs and the optimal k-value higher than Buchanan and Tullock thought. Meanwhile, in terms of decision costs, some rapid developments of ICTs can increase or decrease decision and total costs and the optimal k-value compared to those of Buchanan and Tullock’s original model. We handled a case in which technologies make communication smooth or group thinking more extreme. In these situations, we should make a choice of the socially desirable k-majority rule between the two optimal points. This brings us to the dilemma of “public choice before public choice,” because such a (meta) public choice should be decided before a public decision-making rule is decided, which is when the voters are in the veil of uncertainty. As we previously mentioned, public choice theorists, including Buchanan and Tullock, generally believe political choice can be explained entirely by economic analysis. However, if the dilemma exists in reality, they cannot be completely independent of political decision-making.
